# Impact of Music Education on Mental Health of Higher Education Students: Moderating Role of Emotional Intelligence

**DOI:** 10.3389/fpsyg.2022.938090

**Published:** 2022-06-14

**Authors:** Feng Wang, Xiaoning Huang, Sadaf Zeb, Dan Liu, Yue Wang

**Affiliations:** ^1^South China University of Technology, Guangzhou, China; ^2^Guangxi Arts University, Nanning, China; ^3^Department of Professional Psychology, Bahria University, Islamabad, Pakistan; ^4^Public Basic Teaching Department, Guangzhou Traffic and Transportation Vocational School, Guangzhou, China

**Keywords:** music education, emotional intelligence, mental health, China, higher education students

## Abstract

Music education is one of human kind most universal forms of expression and communication, and it can be found in the daily lives of people of all ages and cultures all over the world. As university life is a time when students are exposed to a great deal of stress, it can have a negative impact on their mental health. Therefore, it is critical to intervene at this stage in their life so that they are prepared to deal with the pressures they will face in the future. The aim of this study was to see how music education affects university students’ mental health, with emotional intelligence functioning as a moderator. The participants in this research were graduate students pursuing degrees in music education. Non probability convenience sampling technique was used to collect and evaluate the data from 265 students studying in different public and private Chinese universities. The data was gathered at a time, and therefore, the study is cross-sectional. The data was collected from January 2022 till the end of March 2022. Many universities have been closed because to COVID-19, therefore data was also gathered online through emails. The data was analyzed quantitatively using the partial least squares (PLS)–structural equation modeling (SEM) technique. The findings backed up the hypotheses. The results revealed that there is a significant effect of music education on student’s mental health. Also, emotional intelligence as a moderator significantly and positively moderates the relationship between music education and students’ mental health. Music has numerous physiological aspects, and listening to it on a daily basis may be beneficial to your general health and well-being. Furthermore, musicians and music students with a high level of emotional intelligence have a better chance of not just performing well in school, college and university or in the music industry, but also of maintaining mental health and improving it.

## Introduction

Music is one of the most universal means of human expression and communication, and it is present in the daily lives of people of all ages and cultures across the globe (Angel-Alvarado et al., 2022; Váradi, 2022). Listening to music, singing, playing (informally or formally), and creating (exploring, composing, and improvising) are all popular pastimes for the vast majority of people (Liu, 2022). Music is a relaxing pastime in and of itself, but its impact extends far beyond that. These activities not only allow people to express their inner thoughts and feelings, but they can also have a variety of beneficial effects on those who take part (Hasanova, 2021). There is a growing body of empirical and experimental research on the broader benefits of musical activity, and research in music sciences suggests that successful musical engagement can positively affect many aspects of human life, including physical, social, educational, and psychological (cognitive and emotional) dimensions (McConkey and Kuebel, 2021). In Western societies, evidence that university students are susceptible to mental health (MH) problems has sparked rising public concern (Campayo-Muñoz and Cabedo-Mas, 2017; Faulkner, 2022).

Numerous prior research has found prevalence of depression, anxiety, and stress among students in higher education around the world (Hedemann and Frazier, 2017; Payne et al., 2020). Undergraduate psychological morbidity is a neglected public health issue with significant consequences for campus health services and mental policy-making. Undergraduate students must deal with the psychological and psychosocial changes that accompany the growth of a self-sufficient personal life, as well as the academic and social obligations that they face during their university studies and preparation for professional employment (Smith, 2021). As a result, many people consider undergraduate education to be crucial for building systems and intervention strategies that can help prevent or lessen mental illnesses (Kim and Kim, 2018). Music has been used to heal people since ancient times. There have been numerous studies on the benefits of music therapy in the treatment of psychiatric diseases and a variety of other health issues. As a result, compared to other undergraduates, music students may experience lower levels of sadness, anxiety, and stress (Demirbatir et al., 2012).

Music captivates and holds the attention of the listener, stimulating and utilizing many different regions of the brain. Music is tailored to a person’s abilities and can reflect them. Music is a powerful memory enhancer because it arranges time in a way that humans can grasp. There is a lot of evidence that there is a link between musical training and cognitive ability. According to Fujioka et al. (2006), music training has a significant impact on brain rewiring for cognitive tasks. Music engages brain networks related with anticipation, attention, and neural clairvoyance, according to Leaver et al. (2009). According to new research, music training improves mental acuity, particularly in the areas of cognitive, verbal, and emotional intelligence (EI; Kraus and Chandrasekaran, 2010). These research back with our findings that music education (MEU) students had high levels of cognitive liveliness.

## Research Literature

### Social Cognitive Theory

Social cognitive theory helps explain how people work by putting the focus on processes that are interactive. The theory says that cognitive activities play a special role in how people can learn new things about their environments. Individuals could provide a reflection on the theory and show how their own actions and the ideas fit together. By linking this theory to music education, the goal was to find out how important changes are for creating good ways to teach and learn for advanced students. It is critical to have a good theoretical understanding of how learning works when planning curricula and teaching services for students with high academic ability. To explain how people work, social cognitive theory focuses on the way that environment, behavior, and personal factors interact in a dynamic way. This way of thinking about how people connect and work came to be called “triadic reciprocal causation” (Avotra et al., 2021). According to the theory, cognitive processes such as watching others and the environment, thinking about it in light of one’s own thoughts and actions, and changing one’s own self-regulatory functions play an important role (Sun, 2022).

### Music Education, Mental Health, and Emotional Intelligence

Mental well-being refers to an individual’s capability to form and maintain mutually beneficial relationships, as well as their psychological functioning and life pleasure (Faulkner, 2022). Psychological health includes the capacity to maintain a sense of autonomy, self-acceptance, and personal development, as well as a sense of life purpose and self-esteem (Smith, 2021). Maintaining a healthy mind involves more than just treating or preventing mental illness (McGinnis, 2018). The majority of people listen to music at certain point in their lives. This can be done by listening to music or actively participating in musical activities. Affective results are common in this type of involvement. Positive emotional, social, physical, and health consequences, as well as intellectual, artistic, and spiritual outcomes, are all benefits and motives for any music participation.

Likewise, young people experienced that listening to music aids them pass the time, lessen boredom, relax, and forget about their worries. They see music as a multidimensional tool for mood regulation, particularly for managing and enhancing their emotions. They may also use loud music as an appropriate means of expressing their displeasure with their parents. Playing an instrument is said to provide people a sense of accomplishment and confidence, as well as a form of communication, yet it can also lead to frustration if their goals are not met (Angel-Alvarado et al., 2022). Making music in a group with peers deepens musical knowledge and understanding while also developing social and personal abilities, resulting in a high level of personal satisfaction and confidence. While there has been little research on the emotional responses of young children to music, physiological, behavioral, and concentration changes have been observed. Overall, the data suggests that music has a substantial impact on our lives, and that the impact is often affective rather than cerebral, with those who actively participate in generating music reaping the greatest benefits. Adult participation is an extension of engagement with active music creating in childhood at home or at school, according to a number of studies. As a result, at least part of the musical education provided during obligatory schooling should be focused on instilling a love of music (Hallam, 2010).

A growing body of research indicates links between music participation and broad indices of mental health, such as greater well-being or emotional competence, implying that music participation may also be associated with better specific mental health outcomes (Faulkner, 2022). Previous research has linked hours of music practice and self-reported music achievement to improved emotional competence. Similarly, a meta-ethnography of research discovered that music activities improved well-being by enhancing emotional control, self-development, providing respite from problems, and facilitating social relationships (Hedemann and Frazier, 2017). According to a study of 1,000 Australian adults, those who interacted with music, such as singing or dancing with others or attending concerts, reported greater well-being than those who engaged in the same experiences alone or did not engage. As a result, this study provides preliminary evidence that music participation is associated with improved general mental health outcomes in both children and adults, with some variation in results depending on the type of music participation (Gustavson et al., 2021).

Emotional intelligence is a type of social intelligence that allows people to detect their own emotions as well as the emotions of others. Furthermore, emotional intelligence enables people to distinguish between such feelings and make appropriate thinking and action decisions. It is a skill that can be learnt, developed, and enhanced. According to Salovey and Mayer (1990), emotional intelligence involves an “ability to monitor one’s own and others’ moods and emotions, to distinguish among them and to utilize this information to direct one’s thinking and actions.” A related definition adds the “capacity to perceive, express, regulate, and manage emotions in an adaptive manner.” Age, developmental level, and gender have all been demonstrated to influence personal or emotional intelligence (Ran et al., 2021). Moreover, our attitude and perspective on life are influenced by emotional intelligence. It aids in the reduction of anxiety as well as the recovery from depression and mood swings. Those who have a high emotional intelligence score are likely to be happier (Vijayabanu and Menon, 2016).

### Effect of Music Education on Mental Health

Music has existed since ancient times and continues to do so today (Váradi, 2022). It has a natural role in controlling people’s emotions and psyche because it is an emotive art. Music is incredibly appealing, and it has spread to every part of the globe. Its non-traditional expression form causes individuals to unconsciously resonate with music. Students can not only appreciate beauty while listening to music, but they may also reduce tension, cultivate emotions, and improve their personalities (Wang, 2020). Many of the psychological and behavioral issues that graduate students face are caused by daily pressures (Payne et al., 2020). It can also have an impact on deeper aspects of people’s personalities, such as self-confidence. Mood disorders such as depression and anxiety are widespread. When a person is stressed, they may experience a variety of negative consequences. A stressful circumstance, for example, can affect a person’s mental ability to do ongoing duties. The use of music as a therapeutic tool has been documented throughout history. The idea of music, mood, and movement is one hypothesis that attempts to explain how music affects people’s psychological responses. According to this notion, “music causes a psychological reaction of enhanced mood, which leads to improved health outcomes.”

Music’s various aspects, such as melody, pitch, and harmony, have been demonstrated to generate a variety of emotional responses in listeners. Music is processed in the limbic system of the brain after passing through the auditory cortex, resulting in an emotional reaction (Pan et al., 2021). Music has increasingly been found to provide both physical and mental health advantages, including improvements in cardiovascular health, a link to a reduction in dementia cases in elderly populations, and increases in general mental well-being markers including stress reduction (Williams et al., 2020). By stimulating students’ creativity and association, MEU can boost students’ interests, emotions, and other non-cognitive characteristics, achieving the goal of cultivating inventive personalities in students. Consequently, music education at colleges and universities is an efficient method for assisting students in overcoming their “dysfunctional” personalities. Through music education, students can develop their creative identities and experience a sense of self-worth and self-efficacy, enabling them to overcome the multiple personality flaws generated by numerous negative factors in the current cultural environment (Li, 2021). As a result, based on the reasoning presented above, it is anticipated that:

*H1:* There is a significant effect of music education on mental health.

## Role of Emotional Intelligence as a Moderator

Emotional intelligence was first studied in the early 1900s. Salovey and Mayer described EI as the ability to notice, comprehend, make sense of, and employ emotional cues from others and oneself in order to manage others’ and one’s own cognitive and emotional functioning (Salovey and Mayer, 1990). They classified it as a subclass of “social intelligence,” because it entails being aware of one’s own and others’ emotions. Daniel Goleman did the most groundbreaking study on EI when he published “EI: Why It Can Matter More Than IQ” in 1995, in which he made startling comparisons to IQ while reaffirming the relevance of EI. We learned about the renowned five components of EI from his book, which are Self-awareness, Self-Regulation, Empathy, Social Skills, and Motivation (Cherry and Morin, 2020; Solomon, 2020).

Emotional intelligence was defined by Salovey and Mayer (1990) as a collection of skills for processing a broad range of emotional information, including (a) perceiving and expressing emotions, (b) supporting emotional thinking, (c) understanding and analyzing emotions, and (d) controlling, directing, and regulating emotions. Managing one’s own emotions well, comprehending the emotions of others, and maintaining emotional control are key human adaptation skills. Emotional intelligence is likely to be a crucial component of the psychological profile of young musicians because (a) it is involved in the process of adapting to the demands of educational and concert activities and (b) it is a factor that increases emotional sensitivity during the process of musical expression. Higher emotional intelligence levels are connected with a greater development of creativity and social competence in collaborative scenarios (in the context of cooperation and teaching). In terms of health, relationships, and overcoming life’s problems, emotional intelligence also increases quality of life (Nogaj, 2020).

Emotional intelligence, according to the creators of the Wong and Law Emotional Intelligence scale, is a skill. It is not, however, as straightforward as that. It is thought to be a construct made up of numerous interrelated dimensions that manifest themselves in diverse ways (Wong and Law, 2002). EI was divided into four dimensions by Salovey and Mayer (1990): Self-Emotional Appraisal refers to a person’s ability to recognize and express one’s own inner emotions in a natural way. Individuals with this talent are able to sense and understand their own emotions in comparison to those of others; Others’ Emotional Appraisal refers to an individual’s ability to comprehend and gain insight into the emotions of those around them. Individuals with these abilities are more sensitive to others’ feelings and have a better understanding of them; Regulation of Emotion in the Self refers to an individual’s ability to control one’s own emotion, allowing them to recover more quickly from any psychological stress; and Use of Emotion to facilitate performance refers to the ability to direct emotion toward useful activities and intimate execution. People believe that acknowledging and properly dealing with emotions adds to their well-being in everyday life. According to a recent meta-analysis, emotional intelligence (EI) is linked to better health (Martins et al., 2010). A further Finnish study (Saarikallio et al., 2015) shows how musical listening affects teenagers’ reported feeling of agency and emotional well-being, while also revealing how context and uniqueness play a role. Aspects of mental health are the subject of an Australian study of young adolescents with depression tendencies (Stewart et al., 2019).

Research on music school students and professional musicians has found that many activities that are based on the needs of music education are also strongly linked to the development of certain psychological traits. Most of these traits have to do with temperaments or personality traits, intellectual and social skills, emotional and motivational traits, and the ability to deal with difficult situations (Yafi et al., 2021). The emotional domain becomes more important because a music student’s ability to handle both school and concert demands is one of the most important parts of how they work. This is when emotional intelligence plays a great role (Nogaj, 2020).

**Figure 1:**
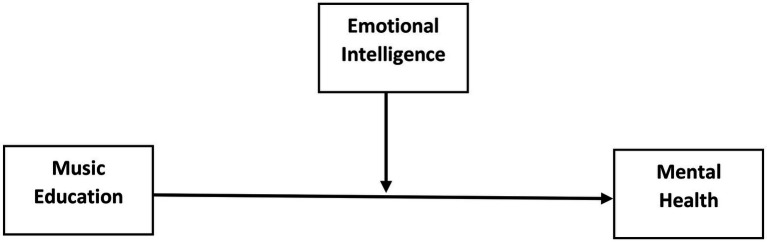
Conceptual model.

The goal of this study was to see how music education affected mental health and what role emotional intelligence played among university students. University is a time when students desire to learn more about themselves physically, psychologically, emotionally, and behaviorally, as well as meet new people, join a social club, or work. They emotionally connect with others while connecting with others. As university life is a time when students are exposed to a great deal of issues regarding academic, personal or emotional area and stressors, these problems can have a negative impact on their mental health. The role of emotional intelligence is critical in dealing with various challenges. Nowadays, every company wants someone with a greater Emotional Quotient (EQ) than an Intelligent Quotient (IQ). When a firm hires people with lower EQ because they have no other option, it is an additional cost for them to implement training programs/sessions to improve Emotional Intelligence (EI). Every young adult would unknowingly overlook “Emotion Well-being” at this stage in their lives, resulting to a chaotic environment. Young individuals must have their emotional intelligence levels assessed since they are more vulnerable to stressful events such as worries in their career and/or personal lives (McGinnis, 2018; Ran et al., 2022). It is critical to provide intervention to young adults who are emotionally inept and lack a strong sense of resilience in order for them to develop into healthy, functional persons. It is critical to intervene at this stage in their life so that they are prepared to deal with the pressures they will face in the future. Giving young adult’s intervention through music makes it much easier for them to connect with their emotions. It was also demonstrated that music has the ability to elicit emotion in the listeners. Many university students experience mental health problems at various stages of their education. This becomes even more important as they near the end of their studies and consider their future options. Market financial situations, particularly as a result of natural disasters such as COVID-19 (Haider et al., 2021), exacerbate the situation for many students. Another important element affecting Chinese university students’ mental health is the pressure to get a job or start their own business. Therefore, the need of the present study was to highlight the effect of music education on higher education institutions students and the use of emotional intelligence strategies by students to manage their emotions and to deal with others as well. Moreover, this study will provide the development of music teaching and its link with mental health issues at university level. Based on the above-mentioned research, a hypothesis has been proposed (see Figure 1):

*H2:* Emotional intelligence plays as a moderating role in the association between music education and mental health.

## Research Methodology

### Sampling and Procedure

The participants in this research were graduate students pursuing degrees in music education. We included both master’s and doctorate students in order to have a better understanding of the diversity of our graduate students. Additionally, since these students often do at least part of their graduate course work simultaneously, interventions happening in classrooms may have an effect on students at both levels. Due to limited resources and inevitable time constraints, it is difficult to collect data from entire population (Taherdoost, 2016). Non-probability convenience sampling technique Stratton (2021), was used to collect and evaluate the data from 265 students studying in different public and private Chinese universities. The data was collected from January 2022 till the end of March 2022. The data was gathered at a time, and therefore, the study is cross-sectional. To maintain confidentiality, the names of the students and universities are not disclosed. The authors contacted the program director and get the permission. Also, 20 universities received an email with the option of either forwarding or providing a list of their graduate students’ email addresses so that we could contact them personally.

The sample size for this research is 265 students, since data was collected during the COVID-19 pandemic and so relied on students’ willingness to complete the questionnaire. Many universities have been closed because to COVID-19, therefore data was also gathered online through emails (i.e., developed questionnaire on Google doc). According to G* power software, the minimum sample size necessary for this research is 107 respondents to achieve a power of 0.95 and a medium size effect of 0.15 (Peng et al., 2012). Though, initially 300 questionnaires were distributed and 279 were received, 14 questionnaires were deleted due missing data and erroneous responses, rendering these questionnaires unreliable and so excluded However, 265 complete questionnaires were received, resulting in an 88.33% response rate. The response rate was quite encouraging in the difficult COVID-19 pandemic situation. The male students are in majority 52.1% and female students were 47.9%. The majority of the samples fell within the ages of 20–30 years 76.2%, 31–40 years were17.2 and 6% were 41–50 years old. According to their education level 44.2% hold bachelor’s degree, 46% Masters and 9.8% Ph.D. holders.

### Measures

The questionnaires used in this study were adapted from those used in previous research. The pilot study was designed to ensure that the questionnaire was accurate before conducting a larger-scale study. There are a total of 80 questions in the survey. Music education was examined using an independent variable of a 20-items scale derived from Sims and Cassidy (2020), with a Cronbach alpha (*α*) reliability of 0.870. A 6-items scale adopted from Koops and Kuebel (2021) with a Cronbach alpha value of 0.906 was utilized to examine the dependent variable mental health. Lastly, the moderating variable Emotional intelligence adopted from Nogaj (2020) based on 54-items scale divided into four sub dimensions: [Acceptance (15-items) with *α* = 0.819 Empathy (18-items) with *α* = 0.777, Control (11-items) with *α* = 0.830, Understanding (10-items) with *α* = 0.797]. The five-point Likert scale was used, with 1 indicating (Strongly Disagree), 2 suggesting (Disagree), 3 indicating (Neutral), 4 indicating (Agree), and 5 indicating (Strongly Agree). It is the most effective instrument for information collecting since it makes it possible to acquire quantitative data in an efficient and easy manner. The reliability of latent variables was assessed using the Cronbach alpha coefficient. Across all variables, the Cronbach alpha values were more than 0.70, which indicates that reliability greater than 0.7 is considered to be acceptable (Henseler et al., 2015). The results of the Cronbach alpha test are displayed in the Table 1.

**Table 1:** Measurement model.

Constructs	*α*	rho_A	CR	AVE	Source
Music education	0.870	0.901	0.905	0.625	Sims and Cassidy, 2020
Emotional intelligence					Nogaj, 2020
Acceptance	0.819	0.821	0.881	0.649	
Control	0.830	0.831	0.887	0.663	
Empathy	0.777	0.778	0.857	0.599	
Understanding	0.797	0.849	0.816	0.566	
Mental health	0.906	0.908	0.931	0.731	Koops and Kuebel, 2021

*α, Cronbach alpha; CR, Composite Reliability; AVE, Average Variance Extracted*.

There is a possibility of common method variance (CMV) in the data since the same respondents were employed to gather data for all variables (Tehseen et al., 2017). In spite of the adoption of procedural remedies such as a cover letter to preserve the confidentiality of the respondent’s personal information and explanations of new phrases, the problem of CMV was still present. CMP (Correlation Matrix Procedure), established by Lindell and Whitney (2001), was also used to examine the CMV’s influence through the correlation of latent variables. CMV could not be detected using this method because the correlation between the primary variables was less than 0.90. When it came to CMV, a comprehensive collinearity examination was utilized. In order to provide estimates of the suitability of the sampling distribution and estimates of population standard errors, the data were bootstrapped to a total of 5,000 samples using Smart-Partial Least Squares (PLS) version 3.2.8 software (Sarstedt et al., 2022). This procedure ensured that the sample data properly reflected the population. Among the multivariate fact-based tests were the following: factor loading, convergent validity, discriminant validity as measured by the Heterotrait–Monotrait Ratio (HTMT), and structural equation modeling (SEM) by calculation of coefficient of determination (*R*^2^), explained predictive relevance (*Q*^2^) and effect size (*f*^2^).

## Results

### Measurement Model Analysis

Due to the reflecting-formative nature of the moderating variable emotional intelligence, a two-stage procedure was developed. Initially, the factor loadings of reflective-formative construct indicators (i.e., first order construct) were studied (Duarte and Amaro, 2018). Only items that met the required criteria were retained. Finally, we calculated scores for latent variables associated with all lower order constructs in order to get single items and assess the reflective-formative construct’s validity (i.e., second order construct). Convergent validity occurs when one item in a construct is linked to other items in the same construct. Composite It can be assessed by factor loadings, composite reliability (CR) and average variance extracted (AVE; Hair et al., 2017). Factor loadings must be more than 0.70 in most cases. Items with outer loadings of 0.40–0.70 should be deleted if their removal improves the CR or AVE values (Sarstedt et al., 2022). Table 2 indicated that the measurement model has convergent validity, by the fact that all Cronbach alpha, composite reliability and average variance extracted were larger than the recommended cut-off requirements.

**Table 2:** Discriminant validity through Heterotrait–Monotrait Ratio (HTMT).

Constructs	EI	MEU	MH	MEU*MH
EI				
MEU	0.663			
MH	0.853	0.794		
MEU*EI	0.141	0.193	0.140	

*EI, Emotional intelligence; MEU, Music education; MH, Mental Health*.

Discriminant validity refers to the ability of a concept to identify itself from other constructs in a model (i.e., both constructs are not assessing the same phenomenon; Henseler et al., 2015). Heterotrait–Monotrait Ratio (HTMT) is the average correlation of the indicators across distinct constructs and their associated components models with conceptually identical structures have a threshold level of 0.90 (Ab Hamid et al., 2017), whereas those with unconnected constructs have a threshold level of 0.85 or lower. The values in Table 2 show that not a single one was higher than 0.85. As a result, the discriminant validity has been established.

Second order constructs were examined for multicollinearity of items and importance of outer weights after first order constructs had been shown to be valid and reliable. According to Duarte and Amaro (2018), second-order constructs may be assessed using two stages. Latent constructs scores for the lower-order items were first obtained. In the first step, the latent constructs were tested, and the scores were utilized as EI items. Table 3 presents the results of an evaluation of the EI measurement model, which was conducted in accordance with the recommendations of Duarte and Amaro (2018). Multicollinearity concerns were investigated using inner variance inflation factor (VIF) values (Craney and Surles, 2002). The VIF is used to assess multicollinearity when two or more components of a concept are strongly linked. A multicollinearity problem is indicated by a number larger than 5. Collinearity between the reflective- formative constructs was investigated. A collinearity estimate showed that EI might be predicted by the constructs of EI such as acceptance, empathy, control, and understanding. Table 3 shows that there were no collinearity issues for second order reflective- formative dimensions with VIF values. The outer weight of reflective-formative indicators was used to assess them. In addition, bootstrapping was used to determine the significance of the weights. Indicator weights and significance are shown in Table 3 that acceptance, empathy, control, and understanding were significant.

**Table 3:** Variance inflation factor and outer weight.

Relationship among constructs	Original sample	*M*	SD	*T* values	VIF	*p* values	2.5%	97.5%
ACC → EI	0.296	0.294	0.023	12.863	2.268	0.000	0.247	0.338
CON → EI	0.285	0.283	0.027	10.516	1.248	0.000	0.227	0.334
EMP → EI	0.283	0.280	0.019	15.034	2.332	0.000	0.243	0.317
UND → EI	0.504	0.505	0.032	15.867	1.331	0.000	0.449	0.571

*SD, standard deviation; M, Mean; VIF, variance inflation factor; ACC, acceptance; EMP, empathy; CON, control; UND, understanding; EI, Emotional intelligence*.

### Structural Equation Modeling

**Figure 2:**
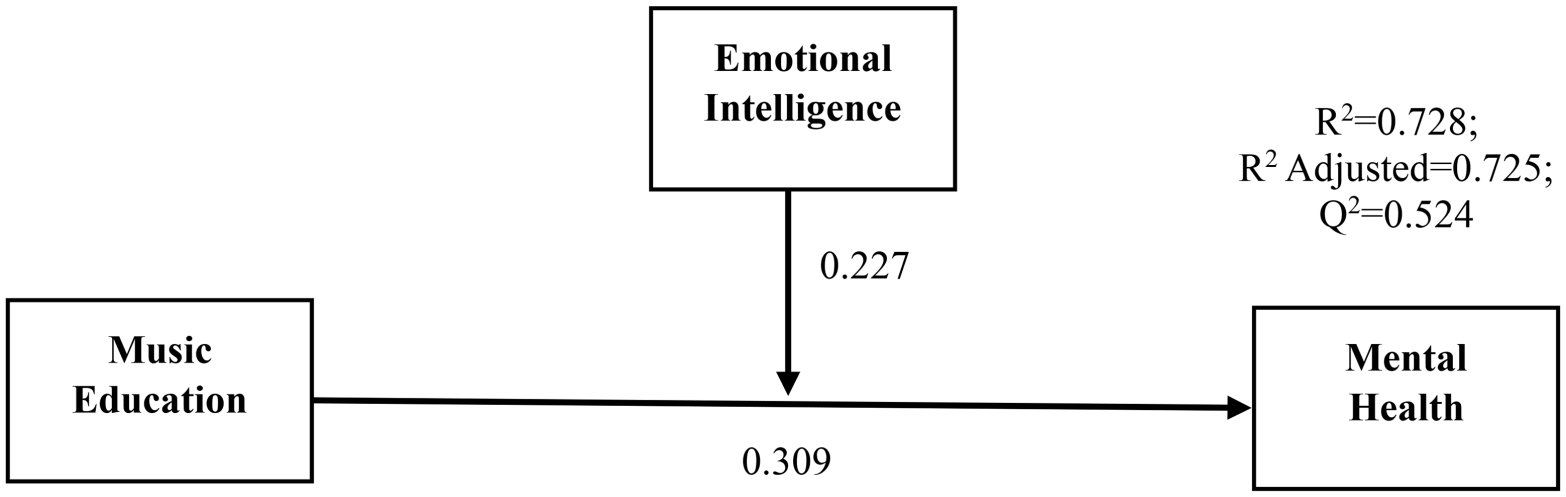
Path analyses, coefficient of determination in the partial least squares (PLS) method.

After analyzing the measurement model, the structural model (also known as the inner model) was evaluated. The inner model depicts the relationship between research components in a theoretical framework. Analysis of a structural model was carried out by evaluating its importance in terms of path coefficients: Coefficient of determination (*R*^2^), Effect size (*f*^2^) and Predictive relevance (*Q*^2^; Sarstedt et al., 2022). However, the multicollinearity of the inner model must be checked first before structural model evaluation since it might mislead the findings. The postulated linkages that link the constructs are known as path coefficients, and they have values ranging from −1 to +1 (Hair et al., 2017). Values close to +1 indicate an extremely favorable association, whereas values close to −1 indicate an extremely negative one. Bootstrapping may be used to determine the relevance of path coefficients in a study. As can be seen in Figure 2, the route coefficients are shown together with the significance and *t*-values associated with them.

As shown in Table 4, the direct effect of music education (MEU) was positively and significantly associated with Mental Health (MH). The path coefficient (*β* = 0.309, *T* = 8.736, *p* < 0.000) demonstrates that a one-unit change in MEU results in a 30.9% change in MH. The Coefficient of Determination (*R*^2^) is a measure of the predictive accuracy of a model. The value of *R*^2^ illustrates the combined influence of exogenous latent variables on endogenous latent variables, and its value ranges from 0 to 1. Higher *R*^2^ values suggested that the model had stronger explanatory ability. According to Cohen (1988), the most significant *R*^2^ values are 0.75, 0.50, and 0.25, which are categorized as large, moderate, and low, respectively. The values of *R*^2^ are shown in Figure 2. The value of *R*^2^ for mental health was 0.728, which is considered large and reflected that 72.8% of variation in mental health was elucidated by music education and emotional intelligence (EI).

**Table 4:** Results of structural equation model.

Hypotheses	Relationship among constructs	*β*	Mean	SD	*T* values	*F* ^2^	*p* values	Remarks
	Direct effect							
H1	MEU → MH	0.309	0.307	0.035	8.736	0.203	0.000	Supported
	Moderating effect							
H2	MEU → EI → MH	0.227	0.226	0.031	4.947	0.144	0.013	Supported

*SD, Standard Deviation; EI, Emotional intelligence; MEU, Music education; MH, Mental Health*.

A measure of the effect size (*f*^2^) is how much the value of *R*^2^ swings when a given exogenous construct is excluded from a model, in order to evaluate whether or not the exclusion has an effect on the endogenous constructs. A value of *f*^2^ greater than 0.35 indicates a large effect size. A result in the range 0.15–0.35 indicates a moderate effect size, while a value between 0.02 and 0.15 indicates a minor effect size (Cohen, 1988). Based on the findings in Table 4, independent construct MEU has a large effect size on MH, whereas the combine MEU (independent construct) and EI (moderator construct) have medium effect size on MH. In order to determine the inner model’s predictive relevance, *Q*^2^ (predictive relevance) is used. *Q*^2^ was determined using the blindfolding method, whereas the omission distance (D) was considered to be 7. A cross-validated redundancy technique was utilized to determine predictive significance. If a number larger than zero indicates predictive relevance, a value less than zero indicates that the model is not predictively relevant (Cohen, 1988). To demonstrate the model’s predictive usefulness, the *Q*^2^ values of endogenous components are shown in Figure 2. Lastly, the moderating effect of emotional intelligence on mental health is also positive and significant (*β* = 0.309, *T* = 8.736, *p* < 0.05). Hence, the results have shown that the hypotheses H1 and H2 were supported.

## Discussion

The study’s goal was to see how music learning affected students’ mental health, with emotional intelligence acting as a moderator. The significance of music education on mental health plays a vital role for students (Faulkner, 2022). Music learning can occur in a variety of settings, including formally (as part of structured lessons in school) and informally (as in the home with family and friends), often non-sequentially and not necessarily intentionally, and where participation in music learning is voluntary rather than mandatory, such as in a community setting. These advantages can be seen throughout life, including in early childhood, youth, and later adulthood (Angel-Alvarado et al., 2022). Music’s contribution to health and well-being has been studied across the lifetime, and evidence of physical and psychological effects has been found. Benefits are also observed in terms of scholastic achievements for young people, and successful musical activity can improve a person’s sense of social inclusion and cohesion (Welch et al., 2020). The current study influence on young adults’ emotional intelligence, as well as assisting people in emotionally connecting with music and associating their troublesome issues to music. This allows individuals/participants to gain a better understanding of their own emotional intelligence. It would also assist folks in achieving a sense of equilibrium in their emotional lives.

Two important hypotheses needed to be investigated. The first hypothesis was to look at the impact of music education on university students’ mental health. The results of present study findings revealed that there is a significant effect of music education on mental health of university students. The study’s findings were consistent with earlier research. Prior literature supports the study hypotheses that music is used in disciplines such as music therapy to improve people’s health and well-being. The findings of the study demonstrated that learning music improves mental wellness. Previous research has shown that taking part in musical activities can improve health and well-being in many ways and situations throughout a person’s life. Musical activities, whether they are about listening, being creative or re-creative, or being in a group or on your own, have the potential to be therapeutic, developmental, enriching, and educational, as long as the people who do them find them interesting, meaningful, and successful (Welch et al., 2020).

The second hypothesis was to find the role of emotional intelligence as a moderator, the association between music education and mental health. The results of present study findings showed that emotional intelligence act as moderator between music education and mental health. The study’s findings were consistent with earlier research. Prior researches supports the present study hypotheses. Previous research revealed that music students had significantly higher levels of EI than other students, particularly in terms of accepting, expressing, and acting on their own emotions (Kim and Kim, 2018). These findings indicate that music students are more aware of their emotions, both positive and negative, are better able to articulate their emotions, and can successfully apply their emotional knowledge. Daily practice on a musical instrument, where emotional expression is explored, may have helped the students to accept, express, and use their emotions. Furthermore, emotional intelligence is an integral part of young artists’ creative talents and is a crucial component of creativity (Nogaj, 2020). According to Smith (2021) music has the ability to heal. It also has an impact on our emotional condition. A lot of music intervention research has been done in a clinical setting. Music therapy can aid patients with motor coordination, neurological function, tranquilization or relaxation, pain relief, depression relief, and overall well-being. Another study discovered a link between emotional expression and music intervention as well (Vijayabanu and Menon, 2016). Previous studies have measured emotional intelligence using a variety of criteria such as self-esteem, self-efficacy, culture, personality, and self-confidence (Zeb et al., 2021); however, the current study focuses on improving emotional intelligence through music learning (Vijayabanu and Menon, 2016).

Personality, motivation, and social and cognitive qualities are all linked to emotional intelligence. The relationship between EI and vital aspects of mental health implies that emotional intelligence is important for life success. Musicians and music students who have a high level of emotional intelligence have a better chance of not only doing well in school or in the music industry, but also of maintaining mental wellness and even better health. There’s also evidence that the amount of time spent learning a musical instrument improves emotional intelligence (Nogaj, 2020). A significant amount of study has been conducted on the relationship between music and emotion (Váradi, 2022). Music has been shown to help people regulate their emotions, and the capacity to recognize the emotions represented by music has been linked to a higher level of emotional intelligence (Campayo-Muñoz and Cabedo-Mas, 2017).

### Theoretical and Practical Implications

This study makes an important contribution to the body of literature. As university students face various mental health issues and few research studies focus on this area, there was an urgent need to investigate the role of music education in improving students’ mental health. This study employs social cognitive theory to examine the conceptualized path. The study’s findings show that music education increases students’ self-esteem, which helps to improve their mental health. The study’s main practical implication is the need to increase emotional intelligence development among music students in high school, college, and university, especially in light of research that demonstrate EI is a predictor of academic success. Music psychology can have a big positive impact on one’s physical and mental wellbeing. Instrument playing can promote emotional expression, socializing, and exploration of many therapeutic themes such as conflict, communication, bereavement, and so on. Music can be used to influence one’s mood. Music engages our brain’s neo-cortex, which relaxes us and reduces impulsivity due to its rhythmic and repetitive elements. It is frequently used to enhance or alter our mood.

While there are some benefits to matching music to our mood, it can also keep us depressed, angry, or nervous. A music therapist can play music that corresponds to the client’s current mood and gradually transform the person into a more happy or peaceful state. Music has numerous physiological benefits, and listening to music on a regular basis may be beneficial to your overall health and well-being. It reduces pain by eliciting emotional responses, focusing cognitive attention, and stimulating sensory pathways in the brain that compete with pain pathways. The music appears to help shift focus away from the pain by competing with the brain’s pain circuits. Furthermore, music and visual art education necessitate a diversity of student labor styles, as well as a spectrum of emotions associated with the presenting of a produced piece. In terms of coping techniques and emotional intelligence, the psychological functioning of art school students varies significantly according on their artistic activity. A musician or music student with a high level of emotional intelligence has a greater possibility of not just excelling in school, college, and university, as well as the music industry, but also of maintaining mental health and even better physical health.

### Limitations

The following are the study’s key flaws. To begin with, further research with a diverse sample can be undertaken in the future to guarantee that results are generalizable. Because the study included a self-report questionnaire, the sample could be skewed if students responded in a way that made them appear socially acceptable. Because preceding writing should provide hypothetical establishments to the examination topic one is studying, a lack of previous research studies could be a potential hindrance. In any case, previous investigations on our research topic were insufficient.

### Suggestion for Future Researches

Following are the suggestions for future researchers. Future study incorporates qualitative study with students to explore further in depth. Future researches proceed by looking into the demographics variables, including gender differences. Moreover, future studies should focus on different variables with music among different population.

## Data Availability Statement

The raw data supporting the conclusions of this article will be made available by the authors, without undue reservation.

## Author Contributions

All authors listed have made a substantial, direct, and intellectual contribution to the work and approved it for publication.

## Funding

This work was supported by the Basic Research Project of South China University of Technology, Project No. 2014XMS212 and the Basic Research Project of South China University of Technology, Project No. x2ys/D215267w.

## Conflict of Interest

The authors declare that the research was conducted in the absence of any commercial or financial relationships that could be construed as a potential conflict of interest.

## Publisher’s Note

All claims expressed in this article are solely those of the authors and do not necessarily represent those of their affiliated organizations, or those of the publisher, the editors and the reviewers. Any product that may be evaluated in this article, or claim that may be made by its manufacturer, is not guaranteed or endorsed by the publisher.
